# Metabolomic analysis revealed the edible and extended-application potential of specific *Polygonum multiflorum* tissues

**DOI:** 10.1016/j.heliyon.2024.e25990

**Published:** 2024-02-15

**Authors:** Yudi Xu, Xianju Liu, Yingying Gao, Yan Liu, Sha Chen, Chang Chen, Jintang Cheng, Cong Guo, Qingxia Xu, Jipeng Di, Jun Zhang, An Liu, Jinzhu Jiang

**Affiliations:** a Key Laboratory of Beijing for Identification and Safety Evaluation of Chinese Medicine, Institute of Chinese Materia Medica, China Academy of Chinese Medical Sciences, Beijing, 100700, China; b Beijing Key Laboratory of Grape Sciences and Enology, CAS Key Laboratory of Plant Resources, Institute of Botany, Chinese Academy of Sciences, Beijing, 100093, China

**Keywords:** *Polygonum multiflorum* thunb., Metabolomic analysis, UPLC-Q-TOF-MS/MS, Tissues, Edibleness, Extended application

## Abstract

The diverse applications of various tissues of *Polygonum Multiflorum* (PM) encompass the use of its leaf and bud as tea and vegetables, as well as the utilization of its expanded root tubers and caulis as medicinal substances. However, previous studies in the field of metabolomics have primarily focused on the medicinal properties of PM. In order to investigate the potential for broader applications of other tissues within PM, a metabolomic analysis was conducted for the first time using UPLC-Q-TOF-MS/MS on 15 fresh PM tissues. A total of 231 compounds, including newly discovered compounds such as torosachrysone and dihydro-trihydroxystilbene acid derivatives, were identified within PM. Through clustering analysis, the PM tissues were categorized into edible and medicinal parts, with edible tissues exhibiting higher levels of phenolic acids, organic acids, and flavonoids, while the accumulation of quinones, dianthrones, stilbenes, and xanthones was observed in medicinal tissues. Comparative analysis demonstrated the potential application of discarded tissues, such as unexpanded root tuber (an industrial alternative to expanded root tuber) and young caulis (with edible potential). Moreover, the quantification of representative metabolites indicated that flowers and buds contained significant amounts of flavonoids or phenolic acids, suggesting their potential as functional food. Additionally, the edible portion of PM exhibited a high content of quercitrin, ranging from 0.59 to 10.37 mg/g. These findings serve as a valuable point of reference for the expanded utilization of PM tissues, thereby mitigating resource waste in this plant.

## 1 Introduction

*Polygonum multiflorum* Thunb. (PM), a member of the Polygonaceae family, has been employed as a medicinal herb for over a millennium [1]. It is renowned for its therapeutic attributes, including anti-aging, hepatoprotective, anti-hyperlipidemic, immunomodulatory, antioxidant, and anti-inflammatory effects [2]. The desicated root tuber of PM (PMR), known as He-Shou-Wu in East Asia and *Fo-Ti* in North America, is particularly noteworthy. Furthermore, the lignified caulis (PMC) of PM is extensively utilized in China and recorded in the Chinese Pharmacopoeia. Prior to usage, these plant parts are typically subjected to processing. PMR possesses detoxifying and intestinal moistening properties that alleviate constipation, while its processed derivative provides tonic and anti-aging advantages [3]. Conversely, PMC has exhibited therapeutic applications in sleep disorders through pharmacological and clinical investigations [4]. These functions are closely associated with the characteristic constituents presented in PM, such as stilbenes, quinones, flavonoids, phenolic acids, dianthrones, procyanidins, and xanthones [5].

In addition to its application in medicinal preparations, PM is also utilized in culinary practices. In various regions of China, such as Jiangxi, Taiwan, and Guizhou province, the leaves of PM are commonly dried and fermented for tea, while the fresh buds are harvested and cooked as edible vegetables [[6], [7], [8]]. Notably, fresh PM leaves are rich in antioxidant constituents, particularly flavonoids [9]. However, the flower, young caulis, unexpanded root tuber, and other plant tissues of PM are usually discarded. Consequently, the presence and accumulation of metabolites in these edible and discarded plant parts remain unknown, thereby limiting the comprehensive utilization of PM plants.

Metabolomics has emerged as a robust methodology for investigating the comprehensive profiles of natural products in plants [10]. It has been extensively utilized in studies of PM root tubers, leaves, and caulis to detect various compounds such as flavonoids, stilbenes, quinones, dianthrones, phenolic acids, organic acids, and xanthones [11,12]. Recent comparative studies between PMR and PMC have revealed significant differences in their component profiles [13,14], with a focus on stilbenes, polygoacetophenosides, flavonoids, and anthraquinones. However, these studies have overlooked the analysis of dianthrones, xanthones, and other metabolites. Moreover, the metabolomic profiles of fresh PM tissues were limited and there has been a lack of metabolite comparison of PM edible and medicinal tissues. These research gaps have hindered the further utilization of specific PM tissues.

In this study, an untargeted UPLC-Q-TOF-MS/MS-based metabolomic profiling method was established to investigate the secondary metabolites present in fresh root tubers, caulis, leaves, flowers, and seeds of *P. multiflorum* Thunb. Subsequently, a successful comparative metabolomic analysis was conducted based on semi-quantitative results obtained from the various PM tissues. The semi-quantitative results were validated through absolute quantitative measurements employing 22 commercially available standards. This comparative analysis of metabolites in various tissues of the PM plant serves as a basis for the extensive utilization of PM plants.

## 2 Materials and methods

### 2.1 Plant materials

PM was collected from Chinese herbal medicine planting bases in Yunnan Province, identified as *Polygonum multiflorum* Thunb. by senior engineer Huijin Zhang from China National Botanical Garden. A PM plant was separated into 15 different tissues, which were listed in Table 1. All samples were immediately frozen in liquid nitrogen after collected, grounded to a powder, and lyophilized until a constant weight. Each tissue was properly stored at 4 °C and subsequently analyzed in triplicate.

**Table 1:** Information of the tissues of *Polygonum multiflorum* (PM).

Tissues	Abbreviations
Bud	B
Bark of caulis	BC
Cutting stem	CS
Expanded root tuber	ERT
Flower	F
Xylem of caulis	XC
Lignified caulis with diameter <4 mm	LC(d < 4 mm)
Lignified caulis with diameter ≥4 mm	LC(d ≥ 4 mm)
Mature leaf	ML
Mature petiole	MP
Rachis	R
Seed	S
Unexpanded root tuber	URT
Young caulis	YC
Young leaf	YL

### 2.2 Standards and reagents

Standards were gallic acid, physcion, 2,3,5,4*′*-tetrahydroxystibene-2-*O-β*-D-glucoside, quercitrin, procyanidin B1, procyanidin B2, procyanidin C1, citric acid, caffeic acid, *trans-p*-coumaric acid, protocatechuic acid, polydatin, malic acid, hyperoside, epicatechin, catechin, sucrose, physcion-8-*O-β*-D-glucoside, emodin, emodin-8-*O-β*-D-glucoside, *p*-hydroxybenzaldehyde, with purity over 98% (Table S1). LC/MS grade acetonitrile and formic acid were obtained from Fisher Scientific. Pure distilled water was obtained from Watsons Water and other analytical grade reagents were obtained from Tianjin Fuyu Fine Chemical Co. Ltd.

### 2.3 Sample and standard solution preparation

200 mg dried powder of 15 PM tissues (totally 45 samples) were accurately weighed and ultrasonically extracted at room temperature by 5 mL methanol-water (75%, v/v) for 30 min. After cooled down and weight replenished with the solvent, the samples were centrifuged (Eppendorf 5424R, 12,000 rpm, 10 min, 4 °C). The supernatant was stored at -20 °C until analysis. 22 standards were accurately weighed and dissolved in 75% methanol to prepare standard samples, then diluted to proper concentrations for the detection of linear relationships.

### 2.4 Untargeted UPLC-Q-TOF-MS/MS conditions

Chromatographic separation was carried out on an Agilent 1290 infinity 2 series UPLC system (Agilent, Palo Alto, CA, USA). 2 μL sample solution was injected into a Waters ACQUITY UPLC® BEH C_18_ column (100 mm × 2.1 mm, 1.7 μm) at 40 °C. The mobile phases are (A) water (0.1% formic acid, v/v) and (B) acetonitrile (0.1% formic acid, v/v). The gradient was as follows: 0–8 min, 10–18% B; 8–14 min, 18–22% B; 14–19 min, 22–33% B; 19–22 min, 33–90% B; 22–26 min, 90-10% B; 26–30 min, 10% B, at a flow rate of 0.3 mL/min.

Metabolite analysis was performed on an Agilent 6540 Q-TOF mass spectrometer equipped with an electrospray ionization (ESI) detector (Agilent, Palo Alto, CA, USA). Samples were detected in negative mode with mass range setting at 50–1500 Da for auto-MS/MS scan. The ESI source operation parameters were as follows: drying gas (N_2_) flow rate and temperature at 8.0 L/min and 300 °C; nebulizer, 30 psig; sheath gas flow rate and temperature at 11.0 L/min and 400 °C; capillary, 3500 V; skimmer, 65 V; OCT 1 RF Vpp, 750 V and fragmentary voltage, 120 V. For MS/MS experiments, the collision energy was adjusted at 35 V to optimize signals so that could obtain maximal structural information from the ions of interest. Accurate mass measurements (error < 10 ppm for analytes) were obtained by means of an automated calibrant delivery system.

Equal volume of each sample was mixed together to prepare the quality control (QC) sample, which was used to monitor the reproducibility of mass spectrometric results. The QC sample (2 μL) was inserted into every 9 detected samples during the analysis. The overlap degree of their total ion chromatogram (TIC) graphs was displayed to assess the feasibility of this repeat-test technology. Blank 75% methanol (2 μL) was injected between samples with the same frequency of QC samples to validate the cross-talking effect of inter-sample.

### 2.5 Qualitative and quantitative metabolite analysis

For qualitative analysis, with Auto-MS/MS extraction tool of Agilent Mass-Hunter Workstation software (Version B.04.00), the molecular and fragment ions of PM metabolites were automatically extracted through the mass-to-charge ratio (*m*/*z*). A diagnostic ion filter strategy was established based on the commercial standards, internal compound library (excerpted from published literatures) and public databases such as PubChem (https://pubchem.ncbi.nlm.nih.gov/) and MassBank (http://www.massbank.jp/). With it, metabolites were rapidly identified, checked and classified.

For quantitative analysis, to ensure the accuracy, the mass peaks of the same metabolite in different samples were integrated and corrected according to the retention time and molecular ion. Metabolite semi-quantification was carried out using the peak area of each metabolite. The percentage of each chromatogram peak in samples to the total peak area of the corresponding one in all samples was calculated to represent the relative content of each metabolite. Accurate quantification was carried out with calibration curves of standard compounds. Calibration curves were built with peak area in X axis and compound concentration (μg/mL) in Y axis to obtain linear fitting equations that were validated by fitting degree R^2^ (R^2^ ≥ 0.9990).

### 2.6 Statistical analysis

Hierarchical cluster analysis (HCA), principal component analysis (PCA) and partial least squares-discriminant analysis (PLS-DA) were performed on SIMCA 14.0 software. Heatmap analysis was performed in Hiplot Pro (https://hiplot.com.cn/), a comprehensive web service for biomedical data analysis and visualization.

## 3 Results and discussion

### 3.1 Characterization of metabolites in different PM tissues

Untargeted metabolomic analysis was employed to comprehensively investigate the metabolites present in different PM tissues. To expedite the classification of compounds, a diagnostic ion filtering strategy was established (Fig. 1). After the neutral loss of glycosyls [glucose (−162 Da), rhamnose (−146 Da), and xylose/arabinose (−132 Da)], small molecules [CO_2_ (−44 Da), C_2_H_2_O (−42 Da), and CH_3_ (−15 Da)] and other chemical groups [feruloyl (−176 Da), galloyl (−152 Da) and malonyl (−86 Da)], the compounds, which exhibited consistent fragmentation pathways, were categorized into the same group. By utilizing standard compounds and existing chemical literature, the fragmentation patterns of each compound were elucidated, along with the identification of their characteristic molecular and fragment ions in both MS and MS^2^ spectra [[15], [16], [17]]. A total of 231 compounds were identified in the fresh tissues of PM (Table S2). These identified metabolites were categorized into ten groups, consisting of 47 quinones, 46 stilbenes, 41 phenolic acids, 26 flavonoids, 17 chromones, 10 xanthones, 10 procyanidins, 10 dianthrones, 4 organic acids and 20 other compounds (Fig. 2A).

**Fig. 1:**
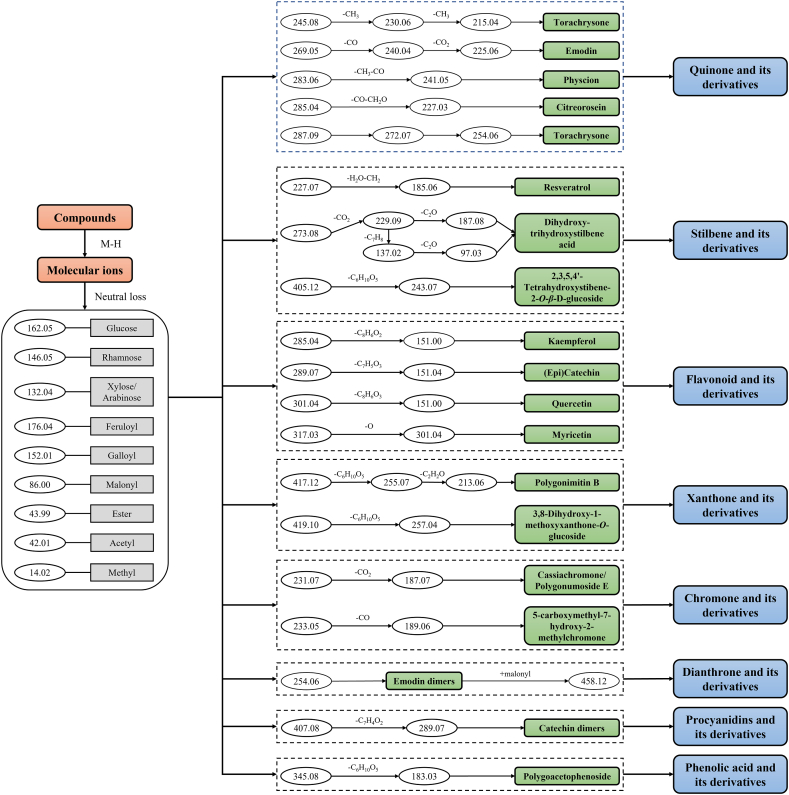
Compound classification method based on diagnostic ion filtering strategy.

**Fig. 2:**
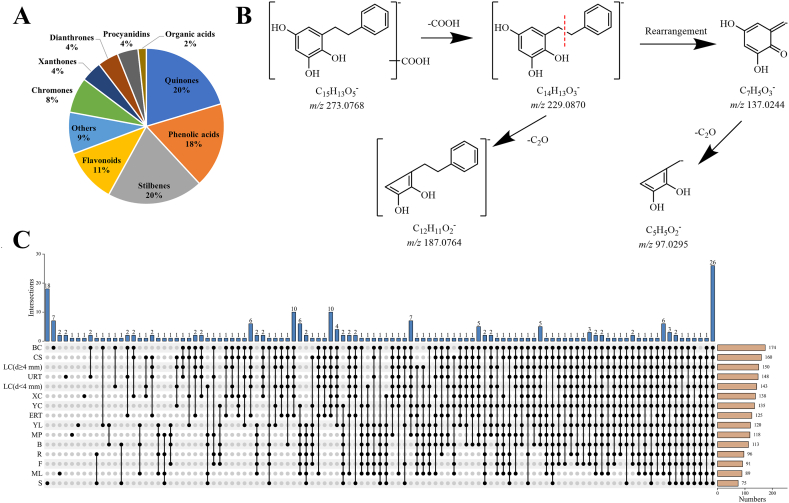
Results of metabolite composition and distribution in different *Polygonum multiflorum* (PM) tissues. A: Composition of the compounds identified in PM (the percentage represented each kind of metabolites in the proportion of total detected metabolites). B: Fragment law of dihydro-trihydroxystilbene acid type metabolites. C: Tissue distribution of PM metabolites. Abbreviations: B: Bud; BC: Bark of caulis; CS: Cutting stem; ERT: expanded root tubers; F: Flowers; XC: Xylem of caulis; LC (d < 4 mm): Lignified caulis (d < 4 mm); LC (d ≥ 4 mm): Lignified caulis (d ≥ 4 mm); ML: Mature leaves; MP: Mature petioles; R: Rachis; S: Seeds; URT: Unexpanded root tubers; YC: Young caulis; YL: Young leaves.

***Quinone-type metabolites*** Quinones, such as naphtoquinones, anthraquinones, anthracyclinones, and others, are a class of compounds that commonly possess an *ortho* or *para* substituted dione conjugated to either an aromatic nucleus (benzoquinones) or a condensed polycyclic aromatic system [18].

Quinones are the significant constituent of PM and exhibit beneficial effects against cancer, developmental anomalies, and tonic tension [19]. The present study primarily identified quinone-type metabolites, including emodin, physcion, citreorosein, torachrysone, and torosachrysone. Emodin, physcion and citreorosein share a common parent nucleus, namely 1,8-dihydroxy-9,10-anthraquinoneas, while torachrysone and torosachrysone possess 1,8-dihydroxy-6-methoxy-naphthalen as their common parent nucleus.

By utilizing commercial standards, it was possible to distinguish emodin derivatives through the [M−H]^-^ ion at *m/z* 269.04, as well as the ions at *m/z* 240.06 and 225.05 which further lost a molecule CO and CO_2_, respectively (Fig. 1). While physcion derivatives can be distinguished by the presence of the [M−H]^-^ ion at *m*/*z* 283.06 and the ion at *m*/*z* 241.05, which is generated through the continuous loss of the neutral molecules CH_3_ and CO (Fig. 1). Furthermore, citreorosein can be differentiated by the ions at *m*/*z* 285.04 and *m*/*z* 227.03, corresponding to [M−H]^-^ and [M-H–CO–CH_2_O]^-^, respectively (Fig. 1).

Torachrysone and torosachrysone are the predominant components found in *Cassia* species cultivars. Torosachrysone, a tetrahydroanthracene precursor of physcion [20], has been identified in PM for the first time. Based on the fragmentation law in references and the commercial standard employed in this study, the typical fragment ions of torachrysone and torosachrysone were promptly categorized (Fig. 1) [21]. The torachrysone derivatives were distinguishable by the [M−H]^-^ ion at *m/z* 245.08, along with the product ions at *m/z* 230.06 and *m/z* 215.04, resulting from the successive loss of two CH_3_ groups (Fig. 1). In the case of torosachrysone, it exhibited an [M−H]^-^ ion at *m/z* 287.09, as well as high abundance ions at *m/z* 272.07, *m/z* 269.08 and *m/z* 254.06, which were in agreement with the findings of a previous study (Fig. 1) [21].

***Stilbene-type metabolites*** Stilbenes are frequently characterized by the presence of two aromatic rings connected by an ethylene bridge [22]. It represents a prominent category of constituents in PM, possessing notable biological activities such as antioxidant, hypolipidemic, antiviral, anti-inflammatory, and anticancer [22].

In PM, a pair of isomers of stilbene, namely *trans-*2,3,5,4*′*-tetrahydroxystilbene and *cis*-2,3,5,4*′*-tetrahydroxystilbene, are significant components. Their glucopyranosides exhibit identical precursor ions at *m/z* 405.12 [M−H]^-^ and product ions at *m/z* 243.07, achieved through the elimination of a glucoside (C_6_H_10_O_5_) (Fig. 1). During the separation process, *cis*-2,3,5,4*′*-tetrahydroxystilbene elutes earlier and exhibits an earlier retention time due to its greater chemical polarity compared to *trans-*2,3,5,4*′*-tetrahydroxystilbene. Resveratrol, a type of stilbene, can be classified based on fragment ions at *m/z* 227.07 and *m/z* 185.06. The ion at *m/z* 227.07 corresponds to the [M−H]^-^ ion of 3,5,4*′*-trihydroxystilbene (Fig. 1).

In this study, a novel structure of dihydro-trihydroxystilbene acid was identified through the diagnostic ion at *m/z* 273.08, which produced a product ion at *m/z* 229.09 by losing CO_2_ (44 Da) (Fig. 1). The ion at *m/z* 229.09 contained two additional hydrogen atoms compared to the aglycone of resveratrol, indicating the presence of a dihydro-trihydroxystilbene skeleton. Furthermore, this was confirmed by the characteristic ion at *m/z* 137.02, which resulted from rearrangement induced by charge transfer, and *m/z* at 187.08 by losing CO_2_ (Fig. 2B). The observed fragmentation pattern following the loss of carboxyl from ion *m/z* 273.08 is consistent with the presence of dihydro-trihydroxystilbene, as reported in the literature [23].

***Flavonoid-type metabolites*** Flavonoids encompass a diverse group of 4-chromanones, including various subclasses such as flavone and isoflavone. Recent research has highlighted the significant potential of flavonoid compounds in physiological and pharmacological applications. These compounds have been found to contribute to the regulation of reactive oxygen species (ROS) *in vivo*, the inhibition of central neural system degradation, and the prevention of diseases such as diabetes, atherosclerosis, obesity, and hypertension [24].

Four distinct types of flavonoids, namely epicatechin, kaempferol, quercetin, and myricetin, were identified in PM tissues. The identification of epicatechin, catechin, quercetin, and hyperoside was confirmed by comparing their fragment patterns and retention times to established standards (Fig. 1). Epicatechin and catechin were found to be isomers, sharing the same ions at *m/z* 289.07 [M−H]^-^ and *m/z* 151.04 [M-H-C_7_H_5_O_3_]^-^. Kaempferol was distinguished by its [M−H]^-^ ion at *m/z* 285.04, with a characteristic fragment ion at *m/z* 151.00 by losing molecule C_8_H_6_O_2_. Quercetin exhibited the [M−H]^-^ ion at *m/z* 301.04, possessing an additional oxygen atom compared to kaempferol. Consequently, both compounds displayed the identical characteristic ion at *m/z* 151.00 resulting from skeleton fragmentation. Quercetin derivatives encompassed dihydro-quercetin, hyperoside, quercitrin, and their respective derivatives. Myricetin, a bioactive compound present in *Polygonum* species, demonstrated the [M−H]^-^ ion at *m/z* 317.03, featuring an extra oxygen atom in comparison to quercetin and exhibiting susceptibility to the loss of a hydroxy group, thereby generating an ion at *m*/*z* 301.04. (Fig. 1).

***Other type metabolites*** Based on the accessible information, the diagnostic ion filtering strategy encompassed other chemical metabolite types, such as xanthones, chromones, dianthrones, procyanidins and some phenolic acids (Fig. 1).

Xanthones, specifically polygonimitin B and 3,8-dihydroxy-1-methoxyxanthone-*O*-glucoside, were identified as the glucopyranoside forms of ions with *m/z* values of 255.07 and 257.04, respectively. Chromones, on the other hand, encompassed polygonumoside E / cassiachromone (with a precursor ion of *m/z* 231.07) and 5-carboxymethyl-7-hydroxy-2-methylchromone (with a precursor ion of *m/z* 233.05). Subsequently, these compounds exhibited characteristic ions at *m*/*z* 187.07 and *m*/*z* 189.06, respectively, upon the loss of CO2 and CO. Dianthrones present in the PM primarily consisted of emodin dimers, which could be distinguished by their *m*/*z* value of 254.06. If an ion with *m/z* 458.12 was detected, the compounds could be classified as containing a malonyl group. The procyanidins present in PM primarily consisted of catechin dimers and exhibited a diagnostic ion at *m/z* 407.08 and *m/z* 289.07 (resulting from the loss of C_7_H_4_O_2_ from *m/z* 407.08), which was confirmed using available standards. Among the phenolic acids, polygoacetophenoside derivatives were specifically distinguished by the ion *m/z* 345.08, along with a product ion at *m/z* 183.03 resulting from the loss of glucoside (C_6_H_10_O_5_).

### 3.2 Distribution of metabolites in different tissues of PM

The distribution of 231 compounds across various tissues is depicted in Fig. 2C. BC exhibited the highest diversity of compounds, while S displayed the lowest (Fig. 2C). Notably, 26 compounds were found to be present in all 15 tissues analyzed, including *trans-*2,3,5,4*′*-tetrahydroxystibene-2-*O-β*-D-glucoside (stilbenes); emodin and torachrysone (quinones); catechin (flavonoids), procyanidin B1 (procyanidins) and 5-carboxymethyl-7-hydroxy-2-methylchromone (chromones); citric acid and malic acid (organic acids); polygoacetophenoside, gallic acid and *p*-hydroxybenzaldehyde (phenolic acids); and sucrose (Fig. 2C). In the lignified caulis and root tuber of PM, a total of ten compounds were specifically identified, with six of them being stilbenes (Fig. 2C).

Additionally, distinct metabolites were found to accumulate exclusively in specific tissues of PM. For instance, BC exhibited seven specific compounds, four of which were identified as quinones (**PA35**, **Q10**, **Q40**, **Q42**, **Q45**, **O15**, **O16**). The presence of kaempferol and compound **PA34** was solely observed in ML. Compound **Q11**, **PA38,** and **O12** exhibited unique existence in XC, YL, and MP, respectively. Furthermore, URT contained two unique stilbenes, namely tetrahydroxystilbene-*O*-(caffeoyl)-glucoside and tetrahydroxystilbene-*O*-deoxyhexoside. Interestingly, S displayed a total of 18 unique compounds, 12 of which were identified as the derivatives and polymers of dihydro-trihydroxystilbene acid. However, the previous literature on this specific metabolite in PM was constrained, with only reports of its decarboxylate metabolites as the product of phase I metabolic reactions of resveratrol *in vivo* [23]. Additionally, the remaining two dihydro-trihydroxystilbene acid derivatives were exclusively found in S and B. These derivatives may serve as essential nutritional constituents for seed germination and bud formation, or as metabolites involved in stilbene biosynthesis in PM plants. Consequently, the distinct metabolomic profiles observed in different tissues of PM can be attributed to their distinct biological functions.

### 3.3 PCA analysis of metabolites in different tissues of PM

In order to investigate the comprehensive metabolomic distinctions among 15 distinct PM tissues and the level of variability within each group, all samples (including QC samples) underwent PCA analysis and the primary outcomes are presented in Supplementary Fig. 1. The PCA scores scatter plot revealed that the QC samples clustered together, indicating the satisfactory repeatability and reliability of the recorded data and analytical approach. Conversely, BC samples were positioned significantly outside the tolerance ellipse established by Hotelling's T2 (95%), signifying their status as outliers and their potential to influence the separation outcomes. Therefore, subsequent PCA and HCA analyses were conducted following the removal of QC samples and outlier BC data. The PCA analysis results revealed a distinct separation of the S samples from the remaining samples. Additionally, samples within each group exhibited close proximity, indicating a high level of consistency among replicate metabolites (Fig. 3A).

**Fig. 3:**
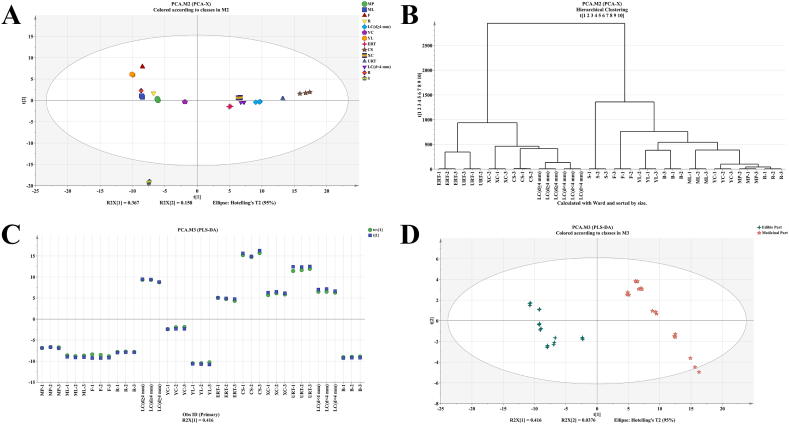
Comparative results of *Polygonum multiflorum* (PM) edible and medicinal part. A, B: Principal component analysis (PCA) and hierarchical cluster analysis (HCA) results of 14 different PM tissues except BC (Bark of caulis, with metabolomic profile far away from the other tissues). C, D: CV-score plot and regular score plot of partial least squares-discriminant analysis (PLS-DA) of 2 p.m. groups. The edible part: B (Buds), F (Flowers), ML (Mature leaves), MP (Mature petioles), R (Rachis), YC (Young caulis), and YL (Young leaves); The medicinal part: CS (Cutting stem), ERT (expanded root tubers), XC (Xylem of caulis), LC (d < 4 mm) (Lignified caulis (d < 4 mm)), LC (d ≥ 4 mm) (Lignified caulis (d ≥ 4 mm)) and URT (Unexpanded root tubers).

Notably, both the PCA scores chart (Fig. 3A) and HCA results (Fig. 3B) demonstrated a clear differentiation between two groups: group 1 comprising F, YL, B, ML, R, MP, and YC, and group 2 consisting of ERT, URT, XC, CS, LC (d ≥ 4 mm), and LC (d < 4 mm). Interestingly, group 1 primarily consisted of edible tissues such as leaves and buds [[6], [7], [8]], while tissues in group 2 are officially utilized as traditional Chinese medicines (TCMs) in clinic. It meant that group 1 possesses potential edibility comparable to the edible portion of PM, while group 2 possesses the medicinal component. The loading plot of PCA (Supplementary Fig. 1B) demonstrated that the number of compounds associated with group 1 was lower than that of group 2, aligning with the findings of the tissue distribution analysis. As a result, the metabolomic profiles exhibit clear distinctions between the medicinal and edible parts of PM.

### 3.4 Metabolite comparison between edible and medicinal part of PM

To elucidate the distinct compounds between the aforementioned PM parts and investigate the potential edibility of specific PM tissues, a PLS-DA analysis was conducted for further differentiation. The modeling process involved the utilization of two principal components. Y-predictive components, which effectively counted for 98.6% (R2 approaching 100%) and 96.7% (Q2 exceeding 50%) of the variation in the training set, as determined through 5-fold cross-validation. These results indicated that the PLS-DA model accurately captured the data and possessed strong predictive capabilities for new data. The CV-scores presented in Fig. 3C indicate that the predicted orthogonal X scores (circle, tcv1) closely align with the actual scores (box, t1). This finding serves as additional evidence for the accuracy of the classification depicted in the regular scores plot (Fig. 3D). In Fig. 3D, it is evident that the medicinal and edible parts of PM are clearly separated from each other in the horizontal direction (t1). In the vertical direction (t2), CS samples deviate from the other samples of the medicinal part but do not significantly impact the clustering of this part.

The Variable Importance for the Projection (VIP) can effectively summarize the importance of the variables in explaining X and correlating with Y. A VIP value greater than 1.00 indicates the variable's significance in explaining X. In PLS-DA model, significant metabolites adhered to the principle of VIP ≥1.00 (Supplementary Fig. 1C), with a significance level of *P* < 0.05 and a fold change of ≥ 2.0 or ≤ 0.5. These selected parameters are displayed in Table S3. A total of 116 metabolites were identified (Supplementary Fig. 1D), including 24 quinones, 22 stilbenes, 16 phenolic acids, 14 flavonoids, 11 chromones, 9 dianthrones, 7 xanthones, 3 procyanidins, 3 organic acids, and 7 others. Among them, six compounds (**PA11**, **PA25**, **PA15**, **F21**, **PA28,** and **PA33**) were specifically present in the edible part, including 5 phenolic acids. Ten compounds (**S26**, **S39**, **S3**, **S2**, **X3**, **S41**, **S5**, **F13**, **C12,** and **S33**) were exclusively distributed in the medicinal part, including seven stilbenes.

To visually analyze the distribution pattern of differential metabolites in the two parts of the plant material, a heatmap analysis was conducted based on the peak area (Fig. 4). The results indicated that both the sample and metabolite content were segregated into two distinct clusters (Fig. 4). The YC samples served as a bridge between the edible and medicinal parts, providing insights into the metabolomic trends of the two parts. From the medicinal part to the edible part, there was an accumulation of phenolic acids, organic acids, and flavonoids, while there was a reduction in quinones, dianthrones, stilbenes, chromones, and xanthones (Fig. 4). The differences in metabolite composition between the edible and medicinal parts of PM were consistent with those observed in the petiole and root of rhubarb [25], and the leaves and roots of *Polygonum cuspidatum* [10]. These plants belong to the same family as PM and are also considered edible medicinal plants (with rhubarb petioles being consumed as vegetables and its roots used as a medicinal resource in European countries; the leaves of *Polygonum cuspidatum* are edible and its roots are medicine).

**Fig. 4:**
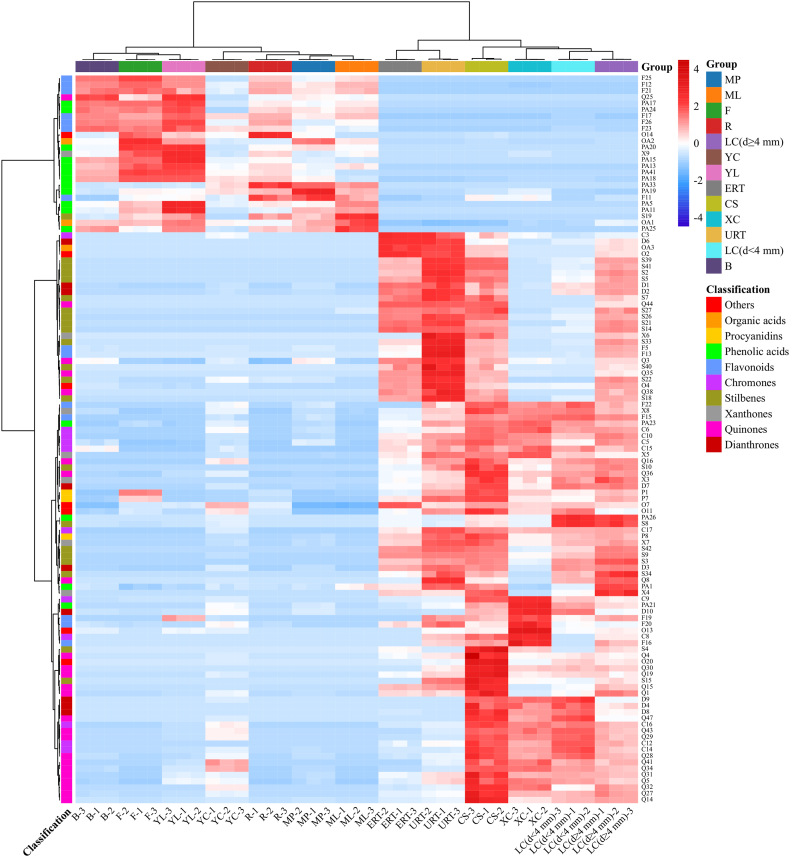
Heatmap analysis of the differential compounds between PM edible and medicinal part. The edible part: B (Buds), F (Flowers), ML (Mature leaves), MP (Mature petioles), R (Rachis), YC (Young caulis), and YL (Young leaves); The medicinal part: CS (Cutting stem), ERT (expanded root tubers), XC (Xylem of caulis), LC (d < 4 mm) (Lignified caulis (d < 4 mm)), LC (d ≥ 4 mm) (Lignified caulis (d ≥ 4 mm)) and URT (Unexpanded root tubers).

The findings of this study also demonstrated the safety of consuming specific PM tissues. The primary constituents of the medicinal part of PM were found to be quinones, stilbenes, dianthrones, and xanthones. These compounds possess various beneficial effects such as anti-aging, immunomodulating, anti-hyperlipidemia, antitumor, hepatoprotective, and anti-inflammatory properties [19]. However, previous studies have indicated that excessive accumulation of quinones and stilbenes may pose a risk of hepatotoxicity, nephrotoxicity, and embryonic toxicity [2]. Notably, dianthrones exhibited significant toxicity against L-02 cell lines, KB tumor cell lines, and zebrafish (*Danio rerio*) embryos *in vivo* [26]. This implies that the use of the medicinal part of PM should be supervised by a clinician to ensure proper and cautious administration. Conversely, the edible part of PM exhibits a high concentration of phenolic acids, organic acids, and flavonoids, which are bioactive compounds known for their antioxidant, anti-inflammatory, and antimicrobial properties. Due to their smaller molecular structures, these compounds can be readily absorbed by the human body. Therefore, the edible part of PM analyzed in this study primarily consisted of these compounds, while exhibiting low dose-dependent side effects due to the low content of quinones, stilbenes, dianthrones, and xanthones.

Hence, the edible portion of PM exhibits significant health benefits and is deemed safer for regular usage. While a direct comparison of bioactivity between the edible and medicinal parts of PM has not been explored, a similar investigation has been conducted on rhubarb. The findings indicate that consuming high doses of rhubarb stalk and root yields comparable antioxidant and anti-inflammatory effects, with the stalk demonstrating lower cell toxicity in comparison to the root [27]. This serves as a valuable reference for the utilization of the edible portion of PM. Consequently, these results reveal that aside from the tissues intended for medicinal purposes, other tissues of PM offer additional potential applications, especially edibleness.

### 3.5 Metabolite comparison of ERT and LC (d ≥ 4 mm)

ERT and LC (d ≥ 4 mm) are two distinct TCMs (PMR and PMC, respectively) with different material compositions. However, previous studies have primarily focused on the analysis of dried forms of these medicines. In this study, we aim to complement the understanding of metabolite differences between the two fresh medicinal tissues. Using the same analytical method as described in section 3.4, a comparison was conducted between ERT and LC (d ≥ 4 mm) (Supplementary Fig. 2). The analysis revealed 97 differential metabolites between these two tissues, encompassing 22 quinones, 16 flavonoids, 16 phenolic acids, 13 chromones, 6 stilbenes, 6 dianthrones, 5 procyanidins, 4 xanthones, 3 organic acids, and 6 other compounds (Supplementary Fig. 2D).

The PMR sample was found to contain higher levels of epicatechin polymers, emodin polymers, emodin, physcion, and cassiachromone derivatives, while the other compounds were more abundant in the PMC sample (Fig. 5). In TCM, raw PMR is known for its ability to moisturize the intestines and alleviate constipation, primarily due to the presence of emodin, physcion, and their derivatives [28]. Furthermore, the presence of epicatechin, its gallate derivatives, and polymers in PMR were found to be positively correlated with the bitterness and astringency of PMR [29], which contribute to its antimalarial properties.

**Fig. 5:**
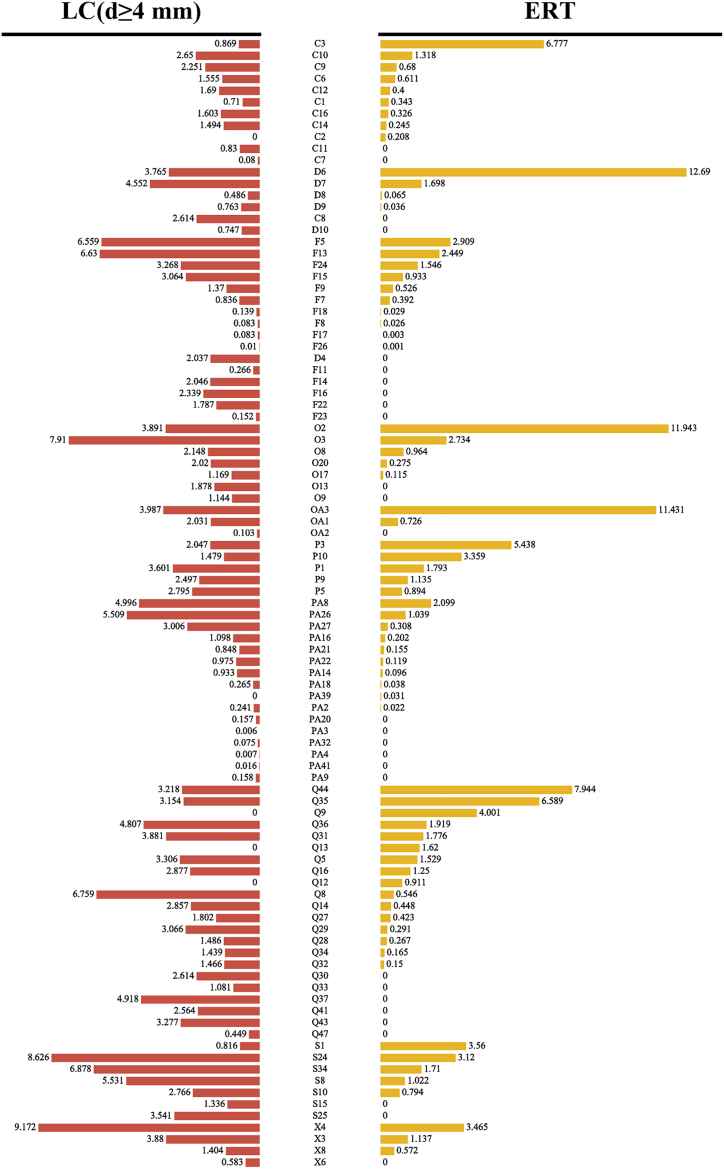
Comparative results of expanded root tuber (ERT) and lignified caulis (d ≥ 4 mm) (LC(d ≥ 4 mm)) from *Polygonum multiflorum* (PM).

In contrast, PMC was found to contain a higher concentration of neuroprotective components such as gallic acid, caffeic acid, protocatechuic acid [30], and some tetrahydroxystibenes [31] so PMC was considered to have the potential to alleviate anxiety (Fig. 5). Furthermore, PMC was found to contain a greater number of metabolites that have not been extensively studied for their pharmaceutical effects, including polygoacetophenoside, torachrysone and torosachrysone derivatives, and dianthrones with malonyl groups (Fig. 5). Consequently, further investigation into the biological activities of these compounds may contribute to a better understanding of the unique functions of PMC. The comparative metabolomic analysis conducted in this research provided insights into the chemical composition of fresh PMR and PMC, shedding light on their distinct therapeutic applications, thereby supplementing the previous studies.

In this study, LC (d ≥ 4 mm) was divided into two distinct parts, namely the bark (referred to as the peripheral part, BC) and the xylem (referred to as the central part, XC). Supplementary Fig. 1A illustrates the spatial distribution of BC and XC, with BC located significantly farther from XC, LC (d ≥ 4 mm), and ERT, while the latter samples were observed to gather in a specific quadrant. This spatial distribution suggests that the metabolomic profile of BC plays a significant role in distinguishing between the two different TCMs, PMR and PMC.

Upon comparing the metabolites present in the bark and xylem of PM caulis (Supplementary Figs. 3A–C), it was observed that BC exhibited a higher abundance and a greater variety of metabolites, with the exception of compound **PA32**, **P10**, **F10**, **PA30**, **Q11,** and **D5** (Supplementary Fig. 3D). This finding suggests that the metabolites in PM caulis are primarily enriched in its bark rather than xylem. In the development of PM, BC and XC have distinct roles, resulting in the accumulation of different substances. XC primarily transports water and mineral salts, with a limited amount of secondary metabolite sufficient to confer decay resistance and reduce permeability in the xylem [32]. In contrast, BC accumulates a significant quantity of phenolic metabolites, which aid in regulating diameter growth, pigmentation, and defending against various pathogens [33].

### 3.6 Metabolite comparison of different caulis and root tuber

Numerous discarded tissues of PM are in varying stages of growth or differ in form from the harvested tissues, such as LC (d ≥ 4 mm) and ERT. In order to explore the extended application potential of the discarded tissues, a comparative analysis was conducted on different caulis and root tubers. Using the same analytical method mentioned in section 3.4, a comparison was made between YC, LC (d < 4 mm), and LC (d ≥ 4 mm). The results of the PLS-DA and model verification can be seen in Fig. 6A–B. By utilizing VIP, *P* value, and fold change, a total of 142 significant metabolites were identified in the caulis of various growth stages. The composition of these metabolites was visually represented in a pie chart (Supplementary Fig. 4A). To effectively depict the clustering of metabolites, a cluster trend graph (Supplementary Fig. 4B) and heatmap (Fig. 6C) were generated. The findings of the study revealed that during the development of PM caulis, its metabolites exhibited a clustering pattern into 8 distinct groups based on their variation law (Supplementary Fig. 4B).

**Fig. 6:**
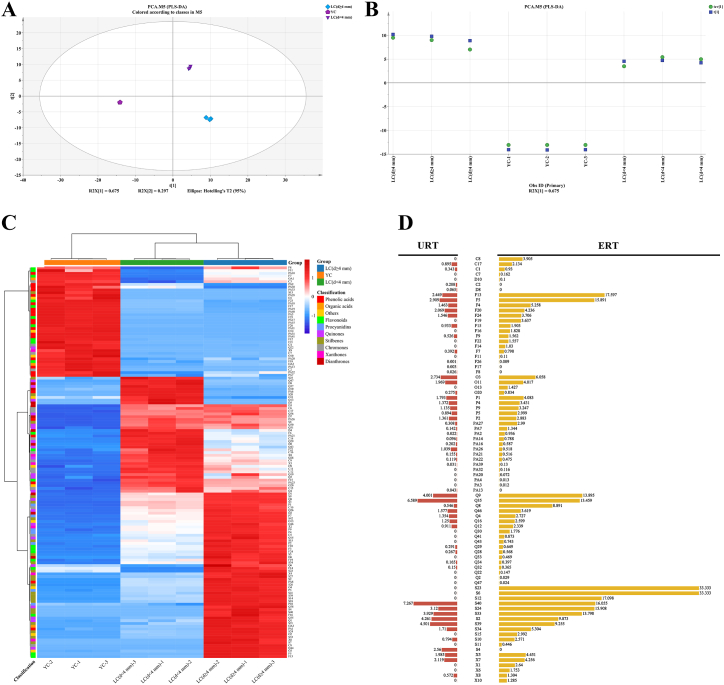
Comparative results of *Polygonum multiflorum* (PM) caulis and root tuber. A, B: PLS-DA analysis and its model verification results of three growth stages of PM caulis: young caulis (YC), lignified caulis (d < 4 mm) (LC (d < 4 mm)) and lignified caulis (d ≥ 4 mm) (LC (d ≥ 4 mm)). C: Heatmap analysis of differential metabolites among three growth stages of PM caulis. D: Content comparative result of the differential metabolites between expanded and unexpanded root tuber (ERT and URT) of PM.

The accumulation patterns of different metabolites were observed to vary, as depicted in Fig. 6C. Specifically, phenolic acids, organic acids, and the majority of flavonoids exhibited the highest abundance in the early stage of development (YC), with their content decreasing as the PM caulis lignified. On the other hand, chromones, dianthrones, and most quinones demonstrated an increase in accumulation from YC to the mid-stage of development [LC (d < 4 mm)], followed by a decrease as the caulis grew thicker and more lignified [LC (d ≥ 4 mm)] (Fig. 6C). The concentration of stilbenes and procyanidins exhibited a gradual increase as the PM caulis grew (Fig. 4C). This observation leads us to hypothesize that flavonoid polymers, specifically procyanidins, are synthesized from flavonoids during the lignification process of the PM caulis, when its diameter reaches or exceeds 4 mm. Conversely, quinone polymers, known as dianthrones, undergo degradation into quinones. The obtained results validate the quality control standard for PM caulis set by the Chinese Pharmacopoeia, which mandates a minimum content of *trans-*2,3,5,4*′*-tetrahydroxystibene-2-*O-β*-D-glucoside at 0.20%. This study further suggests that various compounds, including procyanidins, dianthrones, chromones, and quinones, particularly polygonumoside E and torachrysone derivatives, should be considered as quality evaluation index for PMC.

ERT and URT are two distinct forms of PM root that have been the subject of varying levels of research attention. ERT has received considerable study and recognition for its positive due to its good clinical effectiveness, while URT has been largely overlooked and disregarded. Consequently, conducting a comparative metabolomic analysis of these two forms holds promise for optimizing the utilization of URT. In this study, a total of 83 metabolites were identified as significantly different, primarily encompassing stilbenes, phenolic acids, flavonoids, and quinones (Supplementary Figs. 5A–C). The heatmap (Supplementary Fig. 5D) revealed similar metabolomic profiles between URT and ERT. Furthermore, the content comparison chart (Fig. 6D) revealed that, with the exception of tetrahydroxystilbene-*O*-rhamnoside, which accumulated more in ERT, the majority of compounds exhibited higher levels in URT compared to ERT, often by several fold. This observation suggests that while URT shares a similar metabolomic composition with ERT, it accumulates a greater quantity of compounds. Consequently, URT demonstrates superior utility in the preparation of PM root tuber extract, functional food, and related products, as opposed to ERT.

### 3.7 Quantification of representative metabolites in different PM tissues

To further substantiate the semi-quantitative findings mentioned above and establish the edibility of PM specific tissues and clarify their expanded application, it was necessary to compare the metabolite content of PM tissues and other documented edible plants using absolute quantification. A total of 22 representative compounds, including 7 flavonoids, 5 phenolic acids, 4 quinones, 2 stilbenes, and 2 organic acids, were quantified with precision. The calibration curve parameters can be found in Table S4. Due to the wide range of peak areas observed for some compounds, multiple calibration curves were constructed to ensure accurate measurement within different content ranges (Table S4). Among the compounds measured, kaempferol was only detected in ML with a content of 21.5 ± 0.7 μg/g, and it was not included in Supplementary Fig. 6. The distribution pattern of the other 21 compounds measured (Supplementary Fig. 6) was consistent with the semi-quantitative results mentioned above.

Flavonoids are commonly found in edible medicinal plants and their consumption has been found to be inversely associated with the occurrence of various chronic diseases such as cardiovascular diseases, type II diabetes, neurodegenerative diseases, and cancers [34]. The seven accurately measured flavonoids, including aglycones (catechin and epicatechin), glycosides (hyperoside and quercitrin), and polymers (procyanidin B1, procyanidin B2 and procyanidin C1), which were identified as antioxidants present in nutritional health-care drinks such as tea, coffee and wine [35], as well as edible fruits like apples and *Crataegus pinnatifida* [36,37].

Among the various PM tissues, these flavonoids exhibited the highest concentrations in F, with the exception of procyanidin B1 (Supplementary Figs. 6A–G). Furthermore, the content of these flavonoids in F was consistently higher than that in edible fruits [36,37]. The content of catechin (4.64 mg/g) and quercitrin (10.37 mg/g) in F was found to be consistent with or higher than that of most teas [38]. Previous studies have indicated that these flavonoids play a crucial role in controlling the aroma and taste of tea [39]. This suggests that F possesses significant health benefits and has the potential to be consumed as tea or incorporated into functional food.

Quercitrin, a flavonoid present in various plants, exhibits notable nutritional properties and demonstrates low toxicity. It has been shown to possess bioactivities such as antioxidation, anti-inflammation, anti-microbial effects, immunomodulation, analgesia, wound healing, and vasodilation [40]. The determination results revealed that the concentration of quercitrin in the edible portion of PM was significantly higher (ranging from 0.59 to 10.37 mg/g) compared to the medicinal portion (ranging from 10.71 to 69.79 μg/g) (Supplementary Fig. 6G). In comparison to other Polygonaceae plants known for their quercitrin content, such as buckwheat, *Polygonum aviculare*, and *Polygonum capitatum*, the edible part of PM exhibited a substantially higher concentration of quercitrin [41]. Consequently, the fresh edible portion of PM can be regarded as a promising and novel source of dietary quercitrin.

Additionally, gallic acid, *p-*hydroxybenzaldehyde and *trans-p*-coumaric acid were found to be present in high quantities in S (Supplementary Fig. 6K, N-O). The precursor structures of secondary metabolite biosynthesis in plants, as well as their mothproof and trophic functions, were observed in the study conducted by the Kyoto Encyclopedia of Genes and Genomes (KEGG) pathway database. Additionally, the presence of protocatechuic acid, caffeic acid and quercitrin in B (Supplementary Fig. 6G, L-M) exhibited various health benefits and multiple biological activities [42]. Consequently, B showed great potential as a nutritious food option. Moreover, the accumulation of sucrose in the PM root tuber (Supplementary Fig. 6J) played a significant role in enhancing the starch accumulation in the roots, which served as the fundamental basis for the improved tonic effect during PMR processing. In contrast to the edible portion of PM, the presence of quinones and stilbenes in the medicinal part of PM ensures its therapeutic efficacy (Supplementary Fig. 6P-U).

In comparison to the previous studies on other edible medicinal plants, PM's edible portion contains a higher concentration of health-promoting compounds and fewer or no side effects that are dependent on dosage. These findings provide further support for the regular consumption of PM's flower, bud, leaf, and young caulis, and also serve as supplementary evidence for the aforementioned semi-quantitative results.

## 4 Conclusion

This study presents the novel analysis of metabolomic profiles in 15 distinct fresh tissues of PM, resulting in the identification of 231 compounds. Notably, the identification of torosachrysone and dihydro-trihydroxystilbene acid in PM represents a significant finding. Additionally, clustering analysis successfully categorized the tissues into edible and medicinal parts, highlighting the divergent chemical profiles between these two parts and the two TCMs derived from PM. Furthermore, this study proposes a quality evaluation index for PMC and underscores the potential value of previously discarded tissues, such as URT and YC. Edible tissues exhibited a higher concentration of phenolic acids, organic acids, and flavonoids, whereas medicinal tissues showed a greater accumulation of quinones, dianthrones, stilbenes, and xanthones. The PMR demonstrated a higher presence of epicatechin polymers, emodin polymers, emodin, physcion, and cassiachromone derivatives, while PMC exhibited a higher concentration of other compounds. Proposed quality evaluation indices for PMC included procyanidins, dianthrones, chromones, and quinones, with particular emphasis on polygonumoside E and torachrysone derivatives. URT proved to be more suitable for industrial applications compared to ERT, and YC showed potential as an edible option. Based on the findings of absolute quantification, it was observed that F exhibited promising suitability as a tea or functional food, while the edible portion of PM displayed potential as an alternative source of quercitrin. The metabolite analysis conducted in this study offers valuable insights into the tissue variations within PM plants, thereby providing fundamental data for the expanded utilization of different PM tissues and facilitating future investigations on PM as a whole.

## CRediT authorship contribution statement

**Yudi Xu:** Writing – original draft, Investigation. **Xianju Liu:** Writing – review & editing, Supervision, Project administration. **Yingying Gao:** Validation. **Yan Liu:** Supervision. **Sha Chen:** Methodology. **Chang Chen:** Validation. **Jintang Cheng:** Visualization. **Cong Guo:** Visualization. **Qingxia Xu:** Resources. **Jipeng Di:** Methodology. **Jun Zhang:** Formal analysis. **An Liu:** Supervision, Project administration, Funding acquisition, Conceptualization. **Jinzhu Jiang:** Writing – review & editing, Resources, Project administration, Conceptualization.

## Declaration of competing interest

The authors declare that they have no known competing financial interests or personal relationships that could have appeared to influence the work reported in this paper.

## Figures and Tables

**figs1:**
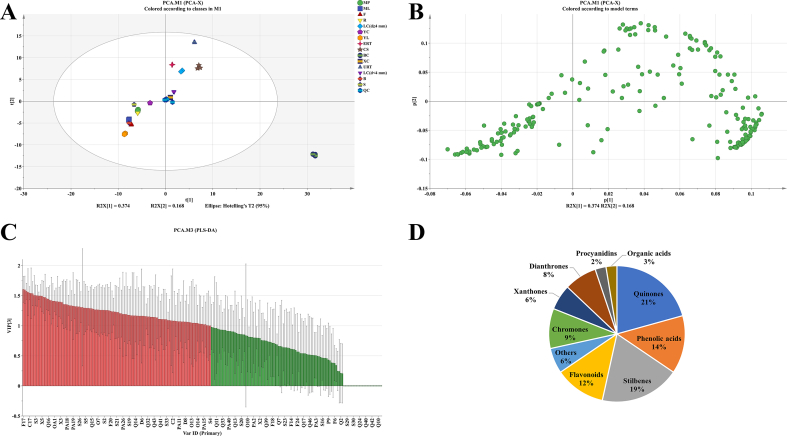


**figs2:**
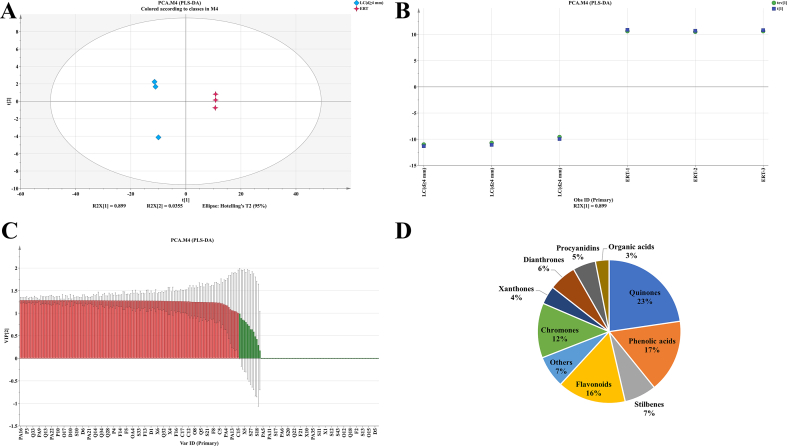


**figs3:**
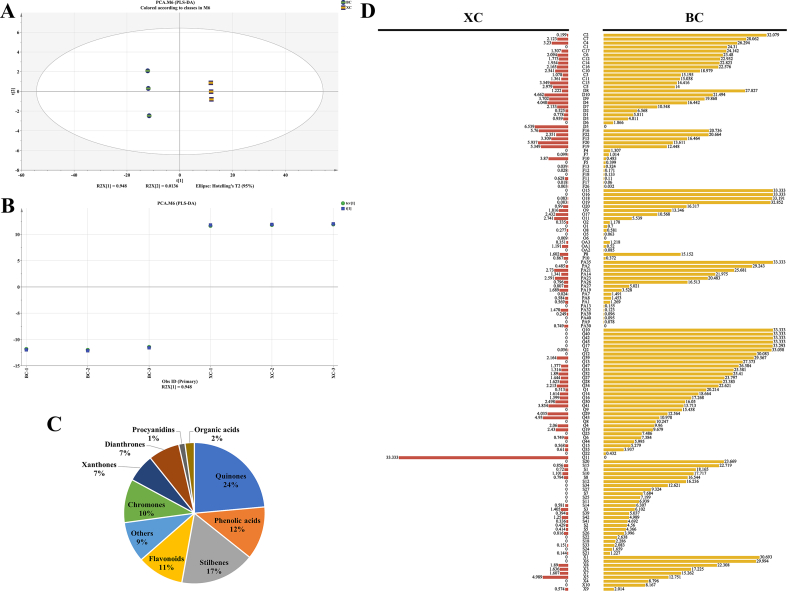


**figs4:**
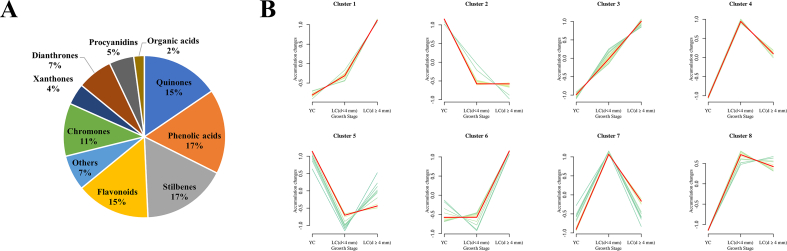


**figs5:**
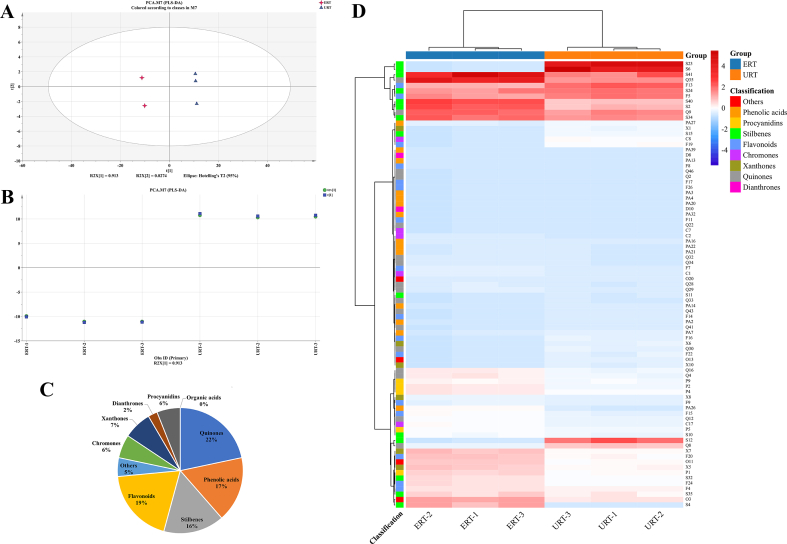


**figs6:**
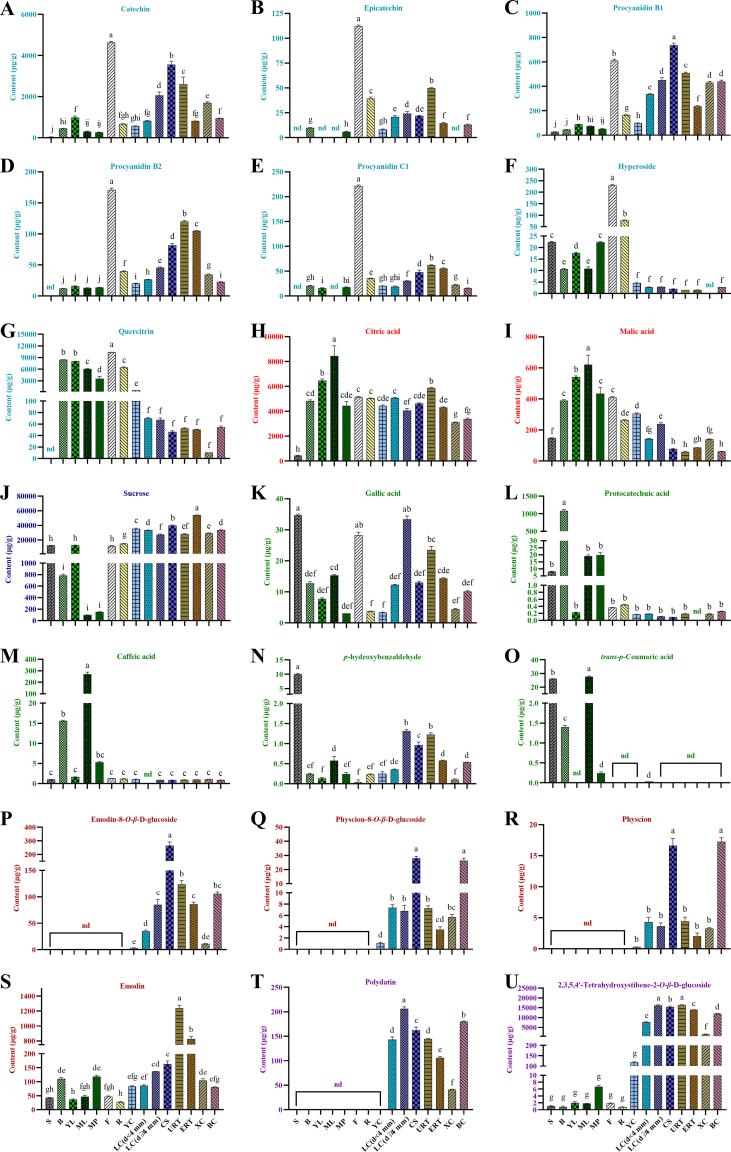


## References

[1] Jian R., Liu Y., Feng J., Li W., Zhan Z. (2023). Herbal textual research on polygoni Multiflori radix and polygoni Multiflori caulis in famous classical formulas, chin. J. Exp. Tradit. Med. Formulae..

[2] Teka T., Wang L., Gao J., Mou J., Pan G., Yu H., Gao X., Han L. (2021). *Polygonum multiflorum*: recent updates on newly isolated compounds, potential hepatotoxic compounds and their mechanisms. J. Ethnopharmacol..

[3] Editorial Committee of Chinese Pharmacopoeia (2020).

[4] Chen Y., Lee C., Huang K., Kuan Y., Chen M. (2015). Prescription patterns of Chinese herbal products for patients with sleep disorder and major depressive disorder in Taiwan. J. Ethnopharmacol..

[5] Qiu X., Zhang J., Huang Z., Zhu D., Xu W. (2013). Profiling of phenolic constituents in *Polygonum multiflorum* Thunb. by combination of ultra-high-pressure liquid chromatography with linear ion trap-Orbitrap mass spectrometry. J. Chromatogr. A..

[6] Cao M., Wu J., Wu L., Gu Z., Xie C., Wu L., Hu J., Xu G. (2022). Separation of three flavonoid glycosides from *Polygonum multiflorum* Thunb. leaves using HSCCC and their antioxidant activities. Eur. Food Res. Technol..

[7] Zhou B. (2015). The key technology of Heshouwu semi-fermented tender leaf tea production. Food Safety Guide.

[8] Zhang H. (2005). Medicinal and vegetable dual-purpose plant-*Polygonum multiflorum*. Rural Practical Technol..

[9] Lee B., Huang Y., Wu S. (2011). Hepatoprotective activity of fresh *Polygonum multiflorum* against HEPG2 hepatocarcinoma cell proliferation. J. Food Drug Anal..

[10] Wu Z., Wang X., Chen M., Hu H., Cao J., Chai T., Wang H. (2019). A study on tissue-specific metabolite variations in *Polygonum cuspidatum* by high-resolution mass spectrometry-based metabolic profiling. Molecules.

[11] Zhang P., Xu Y., Qu F., Zhou P., Zhang J., Bi X., Xiao Y., Liu Y. (2023). Rapid quality evaluation of four kinds of Polygoni Multiflori Radix Praeparata by electronic eye combined with chemometrics. Phytochem. Anal..

[12] Zhao Y., Kao C., Chang Y., Ho Y. (2013). Quality assessment on Polygoni Multiflori Caulis using HPLC/UV/MS combined with principal component analysis. Chem. Cent. J..

[13] Rui W., Xia W., Zhao W., Li B., Li J., Feng Y., Chen H., Zhao S. (2020). Differential constituents in roots, stems and leaves of *Polygonum multiflorum* Thunb. screened by UPLC/ESI-Q-TOF-MS and multivariate statistical analysis. J. Chromatogr. Sci..

[14] Wang G., Shang J., Wu Y., Ding G., Xiao W. (2017). Rapid characterization of the major chemical constituents from Polygoni Multiflori caulis by liquid chromatography tandem mass spectrometry and comparative analysis with Polygoni Multiflori radix. J. Sep. Sci..

[15] Yan Y., Zhang Q., Feng F. (2016). HPLC-TOF‐MS and HPLC-MS/MS combined with multivariate analysis for the characterization and discrimination of phenolic profiles in nonfumigated and sulfur‐fumigated rhubarb. J. Sep. Sci..

[16] Bo R., Wu J., Wu J., Bai L., Ye M., Huang L., Chen H., Rui W. (2021). Rapid analysis and identification of dianthrone glycosides in Polygoni Multiflori Caulis based on enrichment of macroporous absorbent resin and UPLC‐Q‐TOF‐MS/MS. Phytochem. Anal..

[17] Yu Y., Wei X., Liu Y., Dong G., Hao C., Zhang J., Jiang J., Cheng J., Liu A., Chen S. (2022). Identification and quantification of oligomeric proanthocyanidins, alkaloids, and flavonoids in lotus seeds: a potentially rich source of bioactive compounds. Food Chem..

[18] El-Najjar N., Gali-Muhtasib H., Ketola R.A., Vuorela P., Urtti A., Vuorela H. (2011). The chemical and biological activities of quinones: overview and implications in analytical detection. Phytochem. Rev..

[19] Lin L., Ni B., Lin H., Zhang M., Li X., Yin X., Qu C., Ni J. (2015). Traditional usages, botany, phytochemistry, pharmacology and toxicology of *Polygonum multiflorum* Thunb.: a review. J. Ethnopharmacol..

[20] Abdel-Rahman I.A., Beuerle T., Ernst L., Abdel-Baky A.M., Desoky E.E., Ahmed A.S., Beerhues L. (2013). In vitro formation of the anthranoid scaffold by cell-free extracts from yeast-extract-treated Cassia bicapsularis cell cultures. Phytochemistry.

[21] Barberis L., Chevalier W., Toussaint M.L., Binet P., Piola F., Michalet S. (2020). Responses of the species complex Fallopia × bohemica to single-metal contaminations to Cd, Cr or Zn: growth traits, metal accumulation and secondary metabolism. Environ. Monit. Assess..

[22] De Filippis B., Ammazzalorso A., Fantacuzzi M., Giampietro L., Maccallini C., Amoroso R. (2017). Anticancer activity of stilbene-based derivatives. ChemMedChem.

[23] Mei Y., Zhang X., Hu Y., Tong X., Liu W., Chen X., Cao L., Wang Z., Xiao W. (2023). Screening and characterization of xenobiotics in rat bio-samples after oral administration of Shen-Wu-Yi-Shen tablet using UPLC-Q-TOF-MS/MS combined with a targeted and non-targeted strategy. J. Pharm. Biomed. Anal..

[24] de Araújo F.F., de Paulo Farias D., Neri-Numa I.A., Pastore G.M. (2021). Polyphenols and their applications: an approach in food chemistry and innovation potential. Food Chem..

[25] Püssa T., Raudsepp P., Kuzina K., Raal A. (2009). Polyphenolic composition of roots and petioles of Rheum rhaponticum L. Phytochem. Anal..

[26] Yang J., Li W., Liu Y., Wang Q., Cheng X., Wei F., Wang A., Jin H., Ma S. (2018). Acute toxicity screening of different extractions, components and constituents of *Polygonum multiflorum* Thunb. on zebrafish (Danio rerio) embryos in vivo. Biomed. Pharmacother..

[27] Lee H., Yu J., Moon Y. (2022). Antioxidant and anti-inflammatory activities of different parts of rhubarb (Rheum rhabarbarum) compared with da huang root (R. officinale). Kor. J. Food Preservation.

[28] Zhao R., Zhao S., Mao X., Jie F., Liu Z. (2008). Study on the correlation ship between the content of combined anthraquinone and purgative action of steamed *Polygonum multiflorum* Thunb. Lishizhen Med. Mater. Med. Res..

[29] Xing Y., Yan Z., Li Y., Teka T., Pan G., Dou Z., Gao S., He J., Han L. (2021). An effective strategy for distinguishing the processing degree of *Polygonum multiflorum* based on the analysis of substance and taste by LC-MS, ICP-OES and electronic tongue. J. Pharm. Biomed. Anal..

[30] Hornedo-Ortega R., Alvarez-Fernandez M.A., Cerezo A.B., Richard T., Troncoso A.M., Garcia-Parrilla M.C. (2016). Protocatechuic acid: inhibition of fibril formation, destabilization of preformed fibrils of amyloid-*β* and *α*-synuclein, and neuroprotection. J. Agric. Food Chem..

[31] Huang C., Wang Y., Wang J., Yao W., Chen X., Zhang W. (2013). TSG (2,3,4',5-tetrahydroxystilbene 2-*O-β*-D-glucoside) suppresses induction of pro-inflammatory factors by attenuating the binding activity of nuclear factor-*κ*B in microglia. J. Neuroinflammation.

[32] Spicer R. (2005). Senescence in secondary xylem: heartwood formation as an active developmental program. Vascular Transport in Plants.

[33] Popa V.I. (2015). Wood bark as valuable raw material for compounds with biological activity. Tappi J..

[34] Lu M., Xiao Z., Zhang H. (2013). Where do health benefits of flavonoids come from? Insights from flavonoid targets and their evolutionary history. Biochem. Biophys. Res. Commun..

[35] Pintać D., Bekvalac K., Mimica-Dukić N., Rašeta M., Anđelić N., Lesjak M., Orčić D. (2022). Comparison study between popular brands of coffee, tea and red wine regarding polyphenols content and antioxidant activity. Food Chem. Adv..

[36] Liaudanskas M., Viškelis P., Kviklys D., Raudonis R., Janulis V. (2015). A comparative study of phenolic content in apple fruits. Int. J. Food Prop..

[37] Wen L., Guo X., Liu R., You L., Abbasi A.M., Fu X. (2015). Phenolic contents and cellular antioxidant activity of Chinese hawthorn “Crataegus pinnatifida”. Food Chem..

[38] Zhao C., Tang G., Cao S., Xu X., Gan R., Liu Q., Mao Q., Shang A., Li H. (2019). Phenolic profiles and antioxidant activities of 30 tea infusions from green, black, oolong, white, yellow and dark teas. Antioxidants.

[39] Pripdeevech P., Machan T. (2011). Fingerprint of volatile flavour constituents and antioxidant activities of teas from Thailand. Food Chem..

[40] Chen J., Li G., Sun C., Peng F., Yu L., Chen Y., Tan Y., Cao X., Tang Y., Xie X., Peng C. (2022). Chemistry, pharmacokinetics, pharmacological activities, and toxicity of Quercitrin. Phytother Res..

[41] Wu J., Cao M., Zeng J., Xie C., Gu Z., Wu L., Xu G. (2022). Simultaneous determination of three flavonoid glycosides in the leaves of *Polygonum multiflorum* Thunb. By HPLC. Biol. Chem. Eng..

[42] Kakkar S., Bais S. (2014). A review on protocatechuic acid and its pharmacological potential. Int. Scholarly Res. Not..

